# Growth Hormone Improves Growth Retardation Induced by Rapamycin without Blocking Its Antiproliferative and Antiangiogenic Effects on Rat Growth Plate

**DOI:** 10.1371/journal.pone.0034788

**Published:** 2012-04-06

**Authors:** Óscar Álvarez-García, Enrique García-López, Vanessa Loredo, Helena Gil-Peña, Natalia Mejía-Gaviria, Julián Rodríguez-Suárez, Flor Á. Ordóñez, Fernando Santos

**Affiliations:** 1 Department of Pediatrics, University of Oviedo, Oviedo, Spain; 2 Laboratory of Growth and Cancer, Instituto Universitario de Oncología del Principado de Asturias, Oviedo, Asturias, Spain; 3 Department of Pediatrics, Hospital Universitario Central de Asturias, Oviedo, Spain; University of Western Ontario, Canada

## Abstract

Rapamycin, an immunosuppressant agent used in renal transplantation with antitumoral properties, has been reported to impair longitudinal growth in young individuals. As growth hormone (GH) can be used to treat growth retardation in transplanted children, we aimed this study to find out the effect of GH therapy in a model of young rat with growth retardation induced by rapamycin administration. Three groups of 4-week-old rats treated with vehicle (C), daily injections of rapamycin alone (RAPA) or in combination with GH (RGH) at pharmacological doses for 1 week were compared. GH treatment caused a 20% increase in both growth velocity and body length in RGH animals when compared with RAPA group. GH treatment did not increase circulating levels of insulin-like growth factor I, a systemic mediator of GH actions. Instead, GH promoted the maturation and hypertrophy of growth plate chondrocytes, an effect likely related to AKT and ERK1/2 mediated inactivation of GSK3β, increase of glycogen deposits and stabilization of β-catenin. Interestingly, GH did not interfere with the antiproliferative and antiangiogenic activities of rapamycin in the growth plate and did not cause changes in chondrocyte autophagy markers. In summary, these findings indicate that GH administration improves longitudinal growth in rapamycin-treated rats by specifically acting on the process of growth plate chondrocyte hypertrophy but not by counteracting the effects of rapamycin on proliferation and angiogenesis.

## Introduction

Rapamycin or sirolimus (Rapamune, Wyeth Pharmaceuticals, Philadelphia, PA, USA) is used as immunosuppressant in transplanted patients. Rapamycin directly inhibits mammalian target of rapamycin (mTOR), a ubiquitous kinase that controls different pathways related with cell proliferation, growth and metabolism [1]. On the basis of its potent immunosuppressive, antiproliferative and antitumor effects, rapamycin is able to treat multiple conditions simultaneously [2]. Experimental and clinical studies have showed that rapamycin is able not only to provide a low rate of acute rejection and a significant improvement in kidney allograft function, but also to significantly decrease the incidence of posttransplant malignancies [3], [4]. In addition, rapamycin has the potential to interfere with fibrotic processes that often accompany transplant rejection and to influence the preferential development of immunological tolerance [2]. However, the use of rapamycin is associated with many side effects, including increases in serum cholesterol and triglycerides, anemia, proteinuria, skin rashes, retard of wound healing and diarrhea, which can eventually lead to rapamycin withdrawal [5].

In pediatric renal transplantation, rapamycin is mainly used as a substitute for calcineurin inhibitors when nephrotoxicity or proliferative diseases occur [6]. In this group of patients, preservation and improvement of height potential, which can be severely reduced due to multiple factors, is a major challenge [7]. Longitudinal growth is the result of new bone formation during endochondral ossification, in which proliferation and differentiation of epiphyseal growth plate chondrocytes directly correlate with bone elongation. Recent studies from our group found that rapamycin impairs longitudinal growth in young rats, causing marked alterations in the growth plate [8], and that rapamycin disrupts angiogenesis and decreases proliferation and hypertrophy of growth cartilage chondrocytes [9]. In humans, Gonzalez et al [10] reported lower growth rate in a small series of kidney transplanted children treated with rapamycin in comparison with a control group not treated with rapamycin. Therefore, there is growing evidence that rapamycin can adversely affect longitudinal growth this undesirable effect being of special relevance in pediatric renal transplantation.

Over the past two decades, introduction of recombinant human growth hormone (hereafter referred as GH) treatment has been used to improve growth retardation in children with chronic renal disease, including transplanted patients [7]. It is unknown whether the growth promoting effect of GH remains in the presence of rapamycin and, on the contrary, the growth inhibiting effect of rapamycin may be counterbalanced by GH treatment.

The study presented here was designed to find out the effect of GH therapy on longitudinal growth in a model of young rat with growth retardation induced by rapamycin administration.

## Results

### GH Effects on Growth and Nutrition

**Figure 1:**
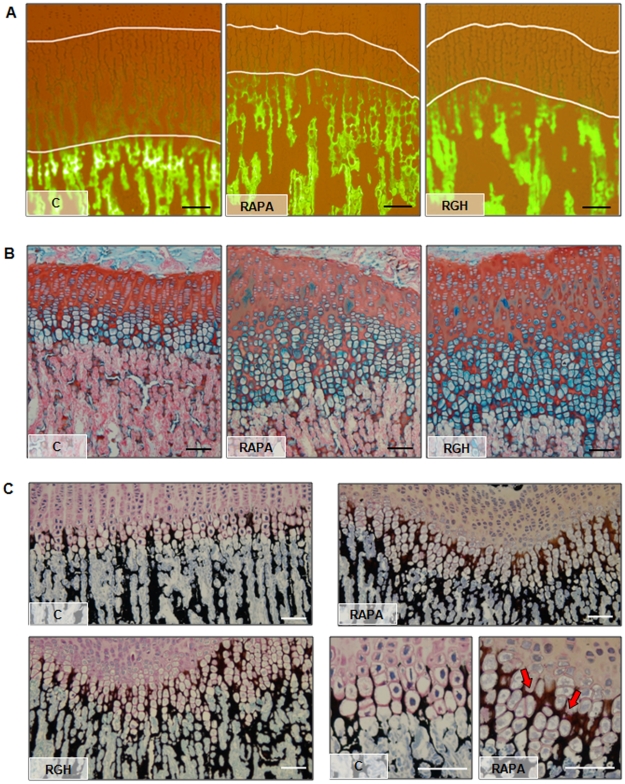
GH effects on longitudinal growth rate and growth plate morphology. (A) Representative images showing the distance between the metaphyseal end of the growth cartilage and the fluorescent calcein front, which indicates longitudinal bone growth rate during the last 3 days of the study, in control rats (C), rats treated with rapamycin (RAPA) or rapamycin and GH (RGH). (B) Representative alcian blue/safranine stained sections showing tibial growth cartilage morphology of C, RAPA and RGH animals. (C) Representative Von Kossa stained sections showing the pattern of extracellular matrix mineralization in proximal tibial growth plate of C, RAPA and RGH animals. Mineralized transverse septa (Red arrows) were often found in RAPA and RGH animals and not in C group. Magnification bars  =  100 µm.

As shown in Table 1, animals treated with rapamycin and GH (RGH) had a significantly greater increase in both nose to tail-tip length gain and longitudinal bone growth rate when compared with those treated with rapamycin (RAPA). Representative images of tibial growth plate sections illustrating the differences in longitudinal bone growth rate are shown in Figure 1A. GH treatment was not associated to higher levels of circulating IGF-I in RGH group when compared with RAPA (C: 288±27 ng/ml; RAPA: 524±32 ng/ml; RGH: 516±93 ng/ml). Trough rapamycin levels were similar in both groups (RAPA: 19.4±4.0 ng/ml *vs* RGH: 18.1±3.8 ng/ml).

**Table 1:** Growth of rats treated with vehicle (C), rapamycin (RAPA) or with rapamycin and growth hormone (RGH).

	C (n = 10)	RAPA (n = 10)	RGH (n = 10)
**Length gain (cm)**	2.4±0.2	2.0±0.2^a^	2.5±0.1^b^
**Longitudinal growth rate (µm/day)**	164±7	82±6^a^	102±5a,b

Values are expressed as mean±SEM.

a Means statistically different from C group (P≤0.05).

b Means statistically different from RAPA group (P≤0.05).

### GH Effects on Growth Plate Morphology

As shown in Figure 1B, RAPA animals exhibited marked morphological alterations in the proliferative and hypertrophic zones of the epiphyseal cartilage, including disorganization of the proliferative layer with loss of the columnar pattern, hardly noticeable transition zone from proliferative to hypertrophic strata, and highly irregular chondro-osseus junction. These alterations were also found in RGH growth plates. In addition, Von Kossa staining revealed an abnormal pattern of mineralization in both groups of rats evidenced by a wide area of extracellular mineralized matrix as well as presence of both transverse bands of mineralized matrix (red arrows) and numerous chondrocytes totally surrounded by mineralized tissue (Figure 1C).

As resumed in Table 2, histomorphometrical analysis revealed that the height of tibial growth cartilage was greater in RGH than in RAPA animals. These differences were also found in the height of the hypertrophic zone. However, the ration of hypertrophic zone to cartilage height was not significantly different between the three groups. Interestingly, the height of the terminal chondrocyte was significantly higher in RGH animals when compared with RAPA.

**Table 2:** Growth plate characteristics of rats treated with vehicle (C), rapamycin (RAPA) or with rapamycin and growth hormone (RGH).

	C (n = 10)	RAPA (n = 10)	RGH (n = 10)
**Epiphyseal cartilage height (µm)**	396±27	471±10^a^	579±40a,b
**Hypertrophic zone height (µm)**	232±21	271±11^a^	345±28a,b
**Terminal chondrocyte height (µm)**	26±1	25±0	27±1^b^

Values are expressed as mean±SEM.

a Means statistically different from C group (P≤0.05).

b Means statistically different from RAPA group (P≤0.05).

**Figure 2:**
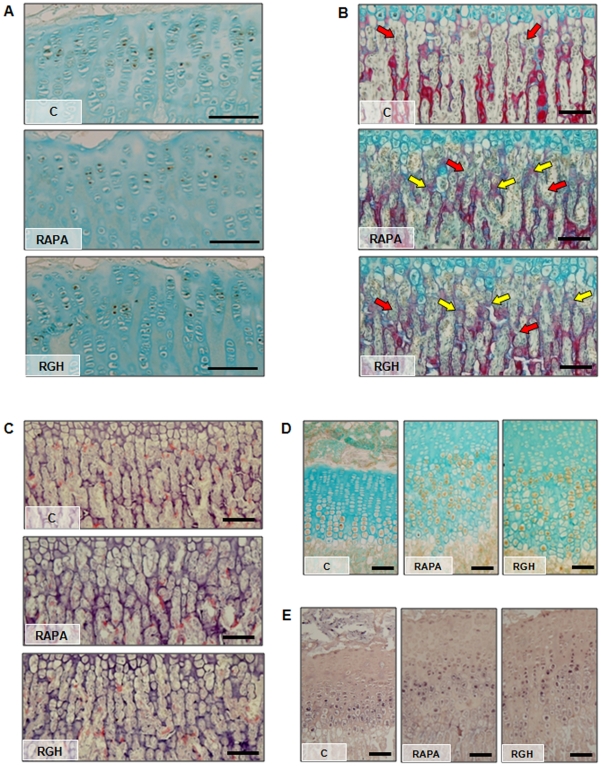
GH effects on growth plate cell proliferation and angiogenesis. (A) Representative images of BrdU immunodetection in the proliferative zone of the epiphyseal cartilage of control rats (C), rats treated with rapamycin (RAPA) or rapamycin and GH (RGH). (B) Representative sections of proximal tibial growth plates stained with picrosirius red/alcian blue showing trabeculae and vascular sprouts arrangement in the primary spongiosa of C, RAPA and RGH animals. Transverse unresorbed septa are indicated with yellow arrows. Vascular sprouts are indicated with red arrows. (C) Representative sections of proximal tibial growth plates stained with tartrate-resistant acid phosphatase (TRAP) showing positive chondroclasts/osteoclasts in the chondro-osseous junction of C, RAPA and RGH animals. Representative images of immunohistochemistry (D) and *in situ* hybridization (E) experiments showing VEGF expression in growth plates of C, RAPA and RGH animals. Magnification bars  =  100 µm.

Finally, chondrocyte proliferation was assessed in growth cartilage by immunodetection of bromodeoxyuridine (BrdU). BrdU-labelled chondrocytes were localized in the proliferative zone in all groups (Figure 2A). Although there were a significant decrease in the percentage of positive cells between rapamycin treated groups and control animals, no statistical differences between RAPA and RGH groups were found [C: 35±1 positive cells/100 cells; RAPA: 28±3 positive cells/100 cells (p≤0.05 *vs* C); RGH: 31±2 positive cells/100 cells (p≤0.05 *vs* C)].

### GH Effects on Growth Plate Angiogenesis

Picrosirius red/alcian blue staining showed morphological anomalies in the primary spongiosa of both groups treated with rapamycin (Figure 2B). In contrast with the typical arrangement of longitudinal trabeculae seen in the primary spongiosa of C rats, numerous transverse unresorbed septa were found in both groups (yellow arrows), strongly depicting the pattern of the invading capillary sprouts (red arrows).

Tartrate-resistant acid phosphatase (TRAP) positive cells were found in the primary spongiosa and along the vascular invasion front in all groups (Figure 2C). In the area between the chondro-osseous junction and 50 mm deep in the primary spongiosa, there was a significant decrease in the number of TRAP-reactive cells per mm^2^ between both groups treated with rapamycin and C animals [C: 262±6; RAPA: 139±21 (p≤0.05 *vs* C); RGH: 103±6 (p≤0.05 *vs* C)].

VEGF immunohistochemical signal was localized within the cytoplasm of hypertrophic chondrocytes from the early hypertrophic zone to the distal end of the cartilage in all groups (Figure 2D). This pattern of VEGF protein distribution was also observed at mRNA level as disclosed by in situ hybridization experiments (Figure 2E). In rapamycin treated animals, it is of note that several chondrocytes adjacent to distal end of the growth cartilage did not express VEGF, despite being metabolically active, as evidenced by normal collagen X expression and alkaline phosphatase activity (see Figure S1). Accordingly, the percentage of terminal VEGF immunopositive cells showed a significant decrease in both rapamycin treated groups when compared with controls [C: 92±1 positive cells/100 terminal chondrocytes; RAPA: 62±4 positive cells/100 terminal chondrocytes (p≤0.05 *vs* C); RGH: 68±3 positive cells/100 terminal chondrocytes (p≤0.05 *vs* C)].

### GH Effects on Chondrocyte Autophagy and Metabolism

**Figure 3:**
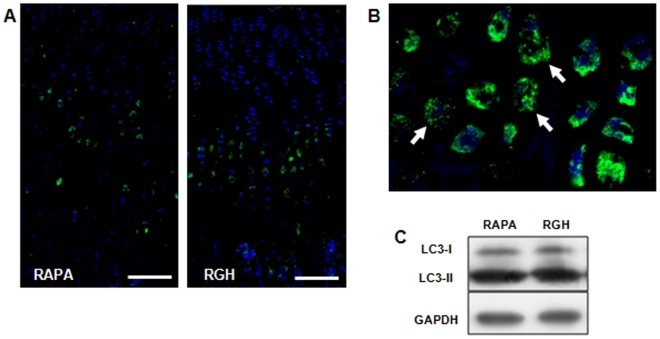
GH effects on chondrocyte autophagy. (A) Immunofluorescent detection of LC3, a marker of autophagy, in the growth plates of rats treated with rapamycin (RAPA) or rapamycin and GH (RGH). Fluorescent signal was observed in prehypertrophic chondrocytes often displaying a punctuate distribution (white arrows in B). (C) Western blot of LC3-I (18 kDa) and LC3-II (16 kDa) in the growth cartilage of rats treated with rapamycin (RAPA) or rapamycin and GH (RGH). GAPDH was used as loading control. Image is representative of three blots giving similar results.

Autophagy was studied in growth cartilage of rapamycin treated rats using two different approaches. First, fluorescent immunodetection of total microtubule-associated protein light chain 3 (LC3) revealed a strong signal mostly in the cytoplasm of early hypertrophic chondrocytes in both groups (Figure 3A). This signal, often detected as punctual staining (arrows in Figure 3B), indicated induction of autophagy in these cells. Second, conversion of LC3-I isoform to LC3-II, as indicative of autophagosome formation, was assessed by western blotting. No differences in LC3-II/LC3-I ratio were found between groups (Figure 3C), suggesting that GH administration did not modify autophagy in the epiphyseal chondrocytes.

**Figure 4:**
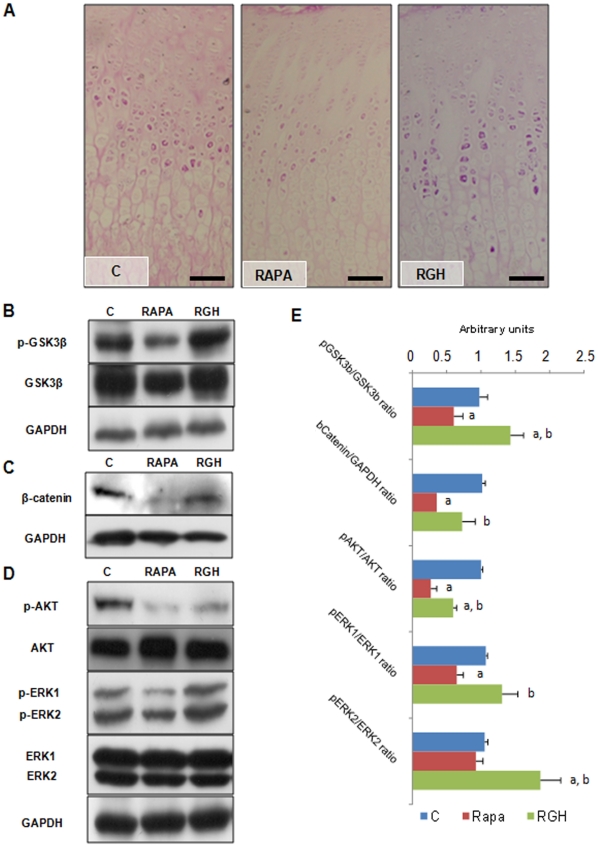
GH effects on chondrocyte metabolism. (A) Periodic acid-Schiff (PAS) reaction in the proximal tibial growth plate of control rats (C), rats treated with rapamycin (RAPA) or rapamycin and GH (RGH). Magnification bars  =  100 µm. (B) Western blot of p-GSK3β (Ser9) and GSK3β in the growth cartilage of C, RAPA and RGH animals. (C) Western blot of β-catenin in the growth cartilage of C, RAPA and RGH animals. (D) Western blot of p-AKT (Thr308), AKT, p-ERK1/2 (Thr202/Tyr204) and ERK1/2 in the growth cartilage of C, RAPA and RGH animals. GAPDH was used as loading control. Images are representative of three blots giving similar results. (E) Densitometry analysis of the western blot experiments shown in B,C and D. At least four animals per group were used per experiment and each assay was repeated at least three times. ^a^Means statistically different from C group (P≤0.05). ^b^Means statistically different from RAPA group (P≤0.05).

Periodic acid-Schiff (PAS) stain was used to identify glycogen deposits, as an index of the energetic reserves of growth plate chondrocytes. As shown in Figure 4A, PAS positive cytoplasmatic granules were mostly seen in chondrocytes of the upper hypertrophic zone, and positively marked cells were gradually disappearing toward the distal end of the cartilage in all groups. In C and RGH samples, PAS-positive granules were more abundant and more intense stained than in RAPA animals suggesting increased glycogen content.

Expression and phosphorylation of several proteins related to glycogen biosynthesis and chondrocyte hypertrophy were studied. IGF-I expression, assessed by immunohistochemistry and in situ hybridization, was confined to the hypertrophic zone of growth cartilage without differences between groups (data not shown). As shown in Figure 4B, GH treatment strongly increased phosphorylation of GSK3β (Ser9), a kinase that inhibits glycogen synthesis and that is inactive upon phosphorylation. In addition, in RGH animals GSK3β inactivation was associated with increased levels of β-catenin (Figure 4C), one of the main GSK3β substrates that is known to be degraded upon phosphorylation. Finally, marked increases of p-AKT (Thr308) and p-ERK1/2 (Thr202/Tyr204) were also found in RGH animals with regard to RAPA group (Figure 4D).

## Discussion

This study shows for the first time that GH administration increases longitudinal growth in young individuals treated with rapamycin at therapeutic doses. We [8], [9] and others [11], [12] have previously reported that rapamycin impairs longitudinal growth in young rats. In the present study, RAPA animals were markedly growth retarded in comparison with control rats. GH treatment caused a 20% increase in both growth velocity and nose to tail tip length in RGH animals when compared with RAPA group. In a recent case report, Rangel et al showed that GH therapy induced catch up growth in a kidney transplanted child with growth failure associated with sirolimus treatment [13]. Taken together, these data suggest that GH administration has the potential to improve longitudinal growth in transplanted children receiving rapamycin. Prospective clinical trials are needed to confirm this assumption.

The present work also shows that GH acts locally in the growth plate to promote linear growth in rapamycin treated animals. GH is known to bind to specific receptors located in the epiphyseal chondrocytes to increase longitudinal growth [14]. In our study, GH treatment did not increase IGF-I circulating levels in RGH animals when compared with RAPA, indicating that GH effects on growth are exerted at local level in the growth plate rather than mediated by an increase of systemic IGF-I. On the other hand, it is unlikely that GH action was mediated by stimulus of the local expression of IGF-I because *in situ* hybridization and immunostaining for IGF-I showed no differences between groups. As we have previously shown [9], IGF-I levels were increased in rapamycin treated animals when compared with control group suggesting a resistance to systemic IGF-I. Since IGF-I levels have been reported to control GH secretion through a negative feedback loop [14], it could be argued that some of rapamycin effects on growth may be the result of lower levels of circulating GH. As GH is secreted in pulsatile form, further studies including repeated serum samples and assessment of the GH profile and the secretory bursts are required to confirm o reject this hypothesis.

An important finding of this study was that GH enhanced chondrocyte hypertrophy in rapamycin treated animals, as disclosed by a significant increase in the height of the terminal chondrocyte. Similar effects on chondrocyte hypertrophy have also been found in uremic young rats receiving GH [15]. Given that chondrocyte enlargement supports nearly 60% of longitudinal bone growth in young rats [16], and height of terminal chondrocytes and longitudinal growth rate have been shown to be positively correlated [17], it is likely that the increase in longitudinal growth seen in RGH animals was mainly produced by terminal chondrocyte enlargement. Although chondrocyte hypertrophy is the major contributor to longitudinal growth [16], numerous aspects of chondrocyte physiology contribute to bone growth, and we cannot rule out that GH could target others than those adversely affected by rapamycin. In fact, GH administration rescued only partially longitudinal growth velocity of rapamycin treated animals, indicating that some rapamycin adverse effects on growth plate biology were still persistent. Indeed, GH administration did not increase chondrocyte proliferation in the growth cartilage and did not normalize the alterations in vascular invasion described in RAPA animals, further supporting this notion.

Chondrocyte hypertrophy is a complex process regulated by largely unknown molecular mechanisms. We have previously shown that mTOR inhibition by rapamycin decreased chondrocyte hypertrophy in the growth plate [8], [9]. Indeed, Akt/mTOR pathway has been found to regulate chondrocyte autophagy [18], a process thought to increase chondrocyte survival and maturation within the epiphyseal cartilage [19]. As shown by immunofluorescent detection of LC3, a major component of autophagosomes and a marker of autophagy [20], we found that chondrocytes in the early hypertrophic cartilage zones exhibited a distinctive autophagic phenotype. This immunofluorescent labeling and the western blot analysis of LC3-II/LC3-I ratio were not different between both groups of animals, indicating that modulation of autophagy is not involved in the effects of GH on growth plate.

**Figure 5:**
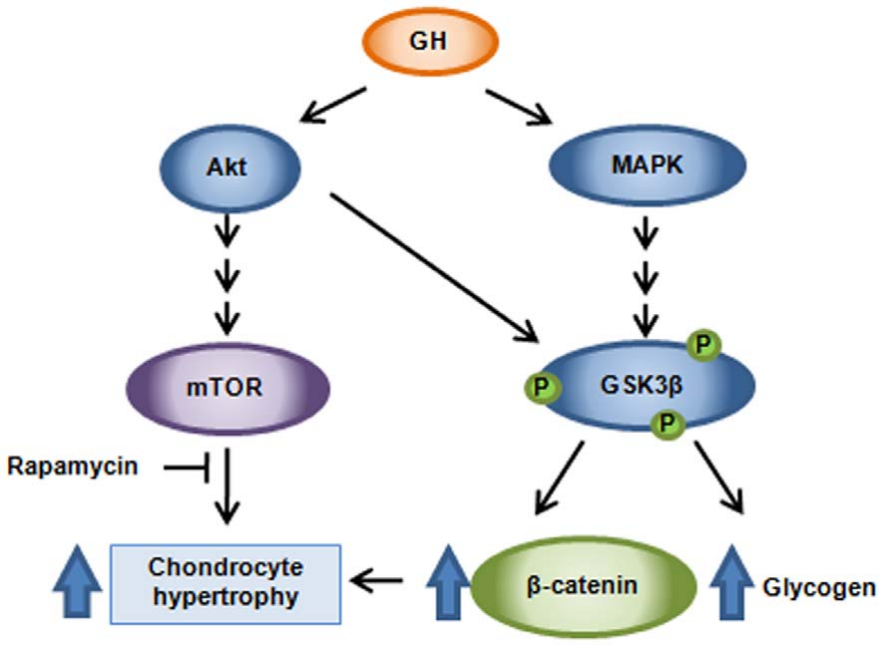
Proposed mechanism of the stimulating effect of GH on the hypertrophy of growth cartilage chondrocytes. GH would signal through PIP3/Akt and MAPK pathways to phosphorylate and inactivate GSK3β in growth plate chondrocytes. This would lead to an increase of glycogen synthesis and stabilization of β-catenin that, eventually, would enhance chondrocyte hypertrophy.

A recent report has showed that hypertrophic differentiation of growth plate chondrocytes during skeletal growth is promoted by phosphorylation and inactivation of GSK-3β [21]. PIP3/Akt and MAPK signalling pathways are both downstream of GH and IGF-I receptors [22], and have been found to directly phosphorylate and inactivate GSK3β [23]. In addition, inactivation of GSK3β upon phosphorylation in Ser9 has been shown to increase glycogen synthesis and to stabilize β-catenin in chondrocytes [21]. β-catenin is a well known mediator of the canonical Wnt signalling pathway, a major promoter of both chondrocyte hypertrophy and final maturation [24]. In our study, we demonstrated that GH administration increases GSK3β phosphorylation, cytoplasmatic glycogen deposits and β-catenin protein levels in growth plate chondrocytes of rapamycin treated rats. Moreover, we also reported a marked increase in Akt and ERK 1/2 phosphorylation in GH treated animals when compared with RAPA group. In the light of these findings, we propose that GH would signal through PIP3/Akt and MAPK pathways to phosphorylate and inactivate GSK3β in epiphyseal chondrocytes (Figure 5). This would lead to an increase of glycogen synthesis and stabilization of β-catenin, which eventually would enhance chondrocyte hypertrophy. However, the role of GSK3 in cartilage physiology is not completely understood. GSK3 protein family consist of two different isoforms, GSK3α and GSK3 β with both overlapping and distinct roles. A recent report by Gillespie *et al.* showed that cartilage-specific inactivation of GSK3β in mice does not affect bone growth [25]. Conversely, the authors report that GSK3α expression levels were increased in these mice suggesting the existence of a compensatory mechanism to counteract the loss of GSK3β in cartilage. We cannot rule out the possibility that GH administration is also inhibiting GSK3α activity in our study, thus resulting in an overall inhibition of GSK3 signalling pathway. In addition, it is of note that activation of the ERK1/2 pathway has been shown to repress endochondral bone growth [26] which is not consistent with the above hypothesis. Further experiments are needed to clarify the role of the various intracellular pathways and its functional interrelationship into the processes of cell growth and maturation.

It is worth to mention that rapamycin antiproliferative and antiangiogenic activities remained in the growth plate of RGH animals. Despite being a well known mitotic agent, GH did not increase cell proliferation in the growth cartilage of RGH animals. In addition, alterations in vascular invasion seen in rapamycin treated animals, such as abnormal arrangement of septa and blood vessels in primary spongiosa, decreased VEGF expression in terminal chondrocytes, and reduction of TRAP positive cells near chondro-osseous junction, were also found in RGH group. Antitumoral effects of rapamycin are thought to be related to inhibition of cell proliferation and angiogenesis. Our study importantly indicates that GH treatment can promote growth without interfering with these effects, at least at the growth plate level. Further studies are needed to clarify whether the potential of GH to increase the risk of malignancy in kidney transplanted children [27] persists in the presence of rapamycin treatment.

In summary, our study reports that GH administration improves longitudinal growth in young rats treated with rapamycin. This GH promoting effect is not related to antagonism of the antiproliferative and antiangiogenic actions of rapamycin, but to an increase in the growth cartilage chondrocyte hypertrophy which involves GSK3β inactivation and β-catenin stabilization.

## Materials and Methods

### Animals

Female Sprague–Dawley rats aged 4 weeks and weighing 70±5 g were housed in individual cages under controlled conditions of light (12 h light/dark cycle) and temperature (21–23°C). All animals received standard rat chow (A03; Panlab, Barcelona, Spain) and tap water. Control animals were fed *ad libitum* and RAPA and RGH were *pair fed* with C group. The study complied with the Directive 86/609/EEC on the protection of Animals used for Experimental and other scientific purposes in the European Union. The protocol was approved by the Ethics Committee on Animal Research of the University of Oviedo, Spain.

### Experimental Protocol and Sample Collection

After 3 days of adaptation to the experimental area, 1 mg/kg/day of rapamycin diluted in 5% dimethyl sulfoxide (DMSO, Sigma, St Louis, MO, USA) was administered intraperitoneally at 10.00 am for seven days to 20 animals whereas control (C, n = 10) rats were injected with 5% DMSO. On day 0 of the protocol, rapamycin treated animals were classified into two groups of 10 individuals each: RAPA and RGH. From day 0 to 6 of the protocol, in addition to rapamycin injection, RGH animals received also 10 IU/kg/day of intraperitoneal recombinant human GH (Norditropin, Novo Nordisk Pharma, Madrid, Spain) at 09.00 and 17.00 h, whereas C and RAPA received vehicle. Animals were killed under anesthesia on day 7 of the protocol. Three days before sacrifice, each animal received 20 mg/kg of calcein (Sigma) by intraperitoneal route for calculation of osseous front advance as an index of longitudinal bone growth rate [9]. BrdU (100 mg/kg; Sigma) was also injected intraperitoneally at 1, 9, and 17 h before animals’ death. Blood samples were collected and stored at −20°C until the measurement of rapamycin and circulating IGF-I concentrations. Proximal ends of right tibiae were immediately frozen in liquid nitrogen for protein expression studies. Proximal ends of left tibiae were embedded in methyl metacrilate as previously described [15]. Samples from half the animals were fixed in 40% ethanol and used for analysis of calcein and BrdU staining. The remaining samples were fixed in 4% paraformaldehyde and used for histomorphometry, immunohistochemistry, immunofluorescence and *in situ* hybridization studies.

### Blood Measurements

Concentrations of rapamycin were measured in whole-blood samples using an Abbott Imx analyzer (Abbott Cientifica, Madrid, Spain) as previously described [8], [9]. IGF-I serum concentrations were measured by enzyme-linked immunosorbent assay using a commercial kit and following manufacturer’s instructions (Immunodiagnostic Systems, Boldon, UK). Inter- and intra-assay variation coefficients were below 7%.

### Growth

Nose to tail-tip length was measured under anesthesia on days 0 and 7. Longitudinal growth rate was measured in 10 µm-thick frontal sections of the proximal end of tibiae as described elsewhere [8].

### Growth Plate Histology and Histomorphometry

Frontal sections (5 mm thick) of proximal tibiae fixed in formalin were stained by the following methods: alcian blue/safranine (Merck, Darmstadt, Germany) for morphometric analysis, alkaline phosphatase stain (Roche, Basel, Switzerland) for chondrocyte maturation, TRAP stain (Sigma) for osteoclast identification, periodic acid-Schiff (PAS) reaction to stain glycogen deposits (Merck), and picrosirius red/alcian blue/hematoxylin (Merck) for analysis of bone and cartilage extracellular matrix. Heights of the growth cartilage and its hypertrophic zone were identified following morphological criteria and measured at regular intervals using an image analysis system previously described [8]. Height of the hypertrophic chondrocytes placed in three most distal rows, those adjacent to the metaphyseal bone, was measured in alternate columns using the same system. Approximately, twenty five measurements were made per section for a total of fifty measurements per animal. The number of TRAP positive cells at the vascular invasion front was measured in an area extending 50 mm from the distal end of the growth cartilage into the primary spongiosa. Two slides per animal were used and results were expressed as the number of positive cells per mm^2^.

### Growth Plate Immunohistochemistry and *in situ* Hybridization

Immunodetection of BrdU, VEGF, IGF-I and collagen X were carried out as described elsewhere [9]. For the immunohistochemical detection of LC3, after overnight incubation with primary antibody (dilution 1∶10, MBL International, Woburn, MA, USA), sections were treated with Alexa Fluor 488–labeled secondary antibody (dilution 1∶500, Fisher-Invitrogen, Barcelona, Spain), counterstained with 4,6-diamidino-2-phenylindole (Vectashield, Vector Laboratories Inc., Peterborough, UK), and examined by confocal microscopy. Two sections per animal were analyzed. The proliferating activity was expressed as the number of positive cells per 100 cells in the proliferative zone, previously defined as the band of tissue between the resting zone and a line traced by the most distal BrdU-labeled cells. VEGF immunohistochemical signal was measured in the three most distal rows of chondrocytes and expressed as number of positive cells per 100 terminal chondrocytes.

In situ hybridization was used to define the mRNA distribution pattern of VEGF and IGF-I in the growth cartilage following a methodology previously described [9].

### Western Blot

A total of 30 µg of protein, extracted from tibial frozen samples as previously described [9], were resolved in SDS-PAGE and transferred to polyvinylidene difluoride membrane (Immobilon-P; Millipore Iberica, Madrid, Spain). After blocking the membranes in 5% (w/v) non-fat dry milk in TBST (Tris-buffered saline, pH 7.4, containing 0.05% Tween 20) at room temperature for 1 h, blots were incubated with primary antibody specific to LC3 (MBL), p-AKT (ser473), AKT, p-ERK 1/2 (Thr202/Tyr204), ERK 1/2, p-GSK3β (ser9), GSK3β, and β-catenin (Cell Signaling, Danvers, MA, USA) diluted 1∶1000 in TBST at 4°C overnight. Immunoblots were then incubated at room temperature for 1 h with the corresponding secondary antibody (Sigma). Peroxidase activity was visualized using a chemiluminescent HRP substrate commercial kit (Immobilon Western; Millipore Iberica) following manufacturer’s instructions.

### Statistical Analysis

Data are given as mean ± SEM. Differences between groups were assessed by ANOVA. Comparisons between two groups were performed by Student t test. A P value ≤0.05 was considered significant. All data sets were analyzed using SPSS 15.0 software package (SPSS, Chicago, IL, USA).

## Supporting Information

Figure S1 **GH effects on chondrocyte expression of collagen type X and alkaline phosphatase activity.** (A) Representative images of immunohistochemistry experiments showing collagen type X expression in growth plates of control rats (C), rats treated with rapamycin (RAPA) or rapamycin and GH (RGH). (B) Representative sections of proximal tibial growth plates showing alkaline phosphatase activity in hypertrophic chondrocytes of C, RAPA and RGH animals.
(TIF)
