# Supplementary figures and images for: Growth Hormone Improves Growth Retardation Induced by Rapamycin without Blocking Its Antiproliferative and Antiangiogenic Effects on Rat Growth Plate
Source: PLoS One. 2012 Apr 6;7(4):e34788. doi: 10.1371/journal.pone.0034788 (PMC3321024; doi:10.1371/journal.pone.0034788)

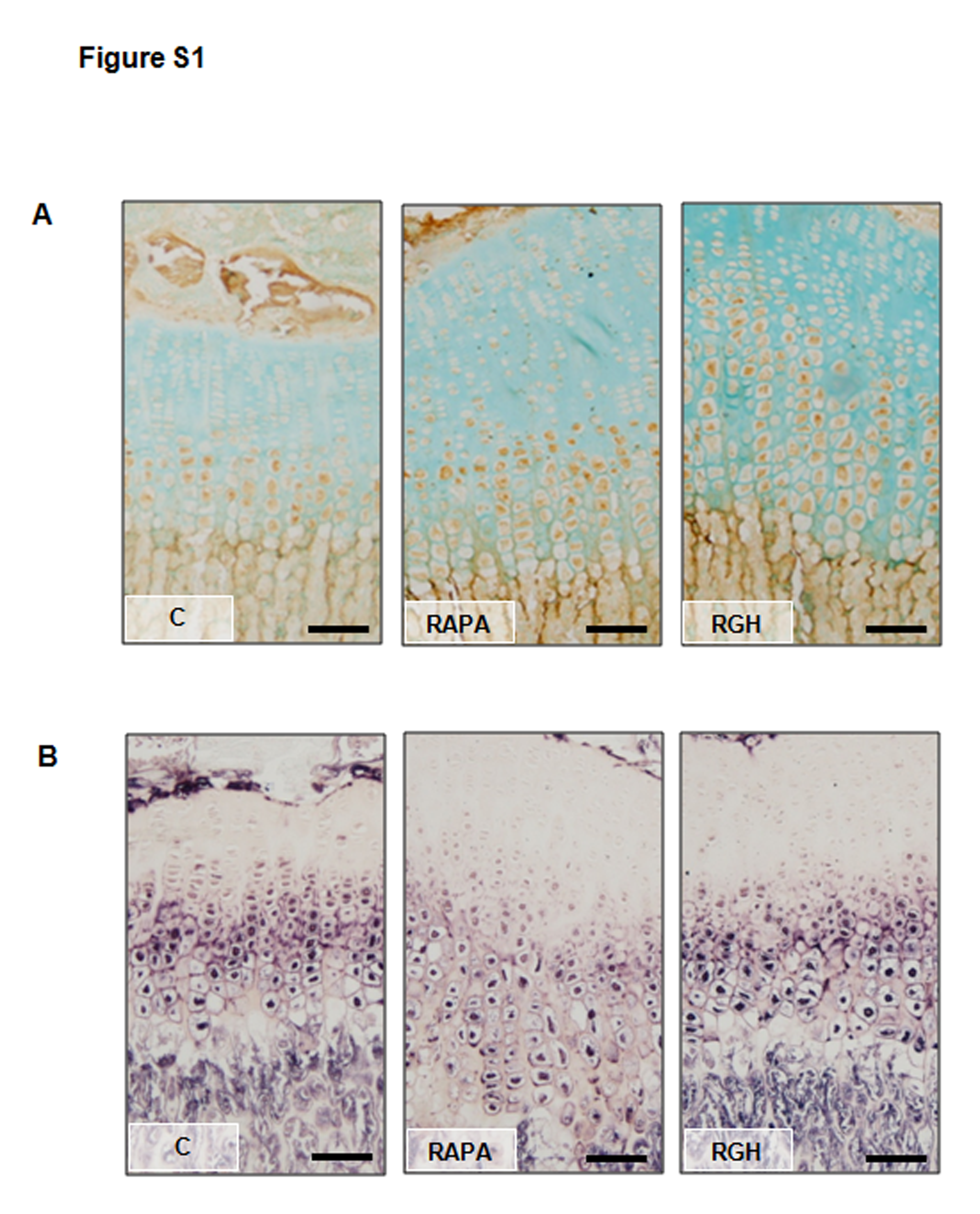

Supplement: Figure S1 — GH effects on chondrocyte expression of collagen type X and alkaline phosphatase activity. (A) Representative images of immunohistochemistry experiments showing collagen type X expression in growth plates of control rats (C), rats treated with rapamycin (RAPA) or rapamycin and GH (RGH). (B) Representative sections of proximal tibial growth plates showing alkaline phosphatase activity in hypertrophic chondrocytes of C, RAPA and RGH animals. (TIF) [file pone.0034788.s001.tif]
